# Comparison of Static versus Dynamic Ultrasound for the Detection of Endotracheal Intubation

**DOI:** 10.5811/westjem.2017.12.36714

**Published:** 2018-02-22

**Authors:** Michael Gottlieb, Damali Nakitende, Tina Sundaram, Anthony Serici, Shital Shah, John Bailitz

**Affiliations:** * Rush University Medical Center, Department of Emergency Medicine, Chicago, Illinois; † Advocate Christ Medical Center, Department of Emergency Medicine, Chicago, Illinois; ‡ Northwestern Memorial Hospital, Department of Emergency Medicine, Chicago, Illinois; § Rush University Medical Center, Department of Health Systems Management, Chicago, Illinois

## Abstract

**Introduction:**

In the emergency department setting, it is essential to rapidly and accurately confirm correct endotracheal tube (ETT) placement. Ultrasound is an increasingly studied modality for identifying ETT location. However, there has been significant variation in techniques between studies, with some using the dynamic technique, while others use a static approach. This study compared the static and dynamic techniques to determine which was more accurate for ETT identification.

**Methods:**

We performed this study in a cadaver lab using three different cadavers to represent variations in neck circumference. Cadavers were randomized to either tracheal or esophageal intubation in equal proportions. Blinded sonographers then assessed the location of the ETT using either static or dynamic sonography. We assessed accuracy of sonographer identification of ETT location, time to identification, and operator confidence.

**Results:**

A total of 120 intubations were performed: 62 tracheal intubations and 58 esophageal intubations. The static technique was 93.6% (95% confidence interval [CI] [84.3% to 98.2%]) sensitive and 98.3% specific (95% CI [90.8% to 99.9%]). The dynamic technique was 92.1% (95% CI [82.4% to 97.4%]) sensitive and 91.2% specific (95% CI [80.7% to 97.1%]). The mean time to identification was 6.72 seconds (95% CI [5.53 to 7.9] seconds) in the static technique and 6.4 seconds (95% CI [5.65 to 7.16] seconds) in the dynamic technique. Operator confidence was 4.9/5.0 (95% CI [4.83 to 4.97]) in the static technique and 4.86/5.0 (95% CI [4.78 to 4.94]) in the dynamic technique. There was no statistically significant difference between groups for any of the outcomes.

**Conclusion:**

This study demonstrated that both the static and dynamic sonography approaches were rapid and accurate for confirming ETT location with no statistically significant difference between modalities. Further studies are recommended to compare these techniques in ED patients and with more novice sonographers.

## INTRODUCTION

Endotracheal intubation is a common procedure in the emergency department (ED). Failure to detect esophageal intubation has the potential for significant morbidity and mortality. Currently, several modalities may be used to detect endotracheal tube (ETT) placement. These often include a combination of auscultation, capnography, or ultrasound. However, there are inherent limitations with each of these methods. The potentially loud environment of the ED can make auscultation difficult, and quantitative capnography is not universally available at all centers.^1^

Ultrasound has been demonstrated to confirm ETT placement rapidly and accurately with recent meta-analyses demonstrating accuracy approaching that of capnography.^2^,^3^ Additionally, ultrasound offers the advantage of directly visualizing the location of the ETT in cases when capnography may be less reliable (eg, cardiac arrest or hypopharyngeal placement).^4^ However, studies have varied in the techniques described, with some using real-time, dynamic confirmation, while others use post-intubation, static imaging.

The goal of this study was to determine whether there was a difference in the accuracy between the static and dynamic approaches when confirming ETT location. Secondary outcomes included time to identification and operator confidence.

## METHODS

This was a blinded, randomized, controlled trial performed in the cadaver lab of an academic hospital located in Chicago, Illinois. Three cadavers with different neck circumferences were used to simulate the variations in live patient populations. Cadaver #1 had a neck circumference of 32 cm, cadaver #2 had a neck circumference of 34 cm, and cadaver #3 had a neck circumference of 37 cm. Local institutional review board approval was obtained for this study with waiver of informed consent. This study was conducted in accordance with the Standards for the Reporting of Diagnostic Accuracy studies (STARD) criteria.^5^

Two attending emergency physicians with extensive intubation experience intubated each cadaver with a size 7.0 ETT using video laryngoscopy. Each cadaver was randomized a priori to either tracheal or esophageal intubation using a random number generator, with the goal of having equivalent numbers of tracheal and esophageal intubations in order to best define the test characteristics of each approach. The video screen was directed away from the sonographers and the intubating providers were instructed to look away after placement to avoid any potential reaction to bias the sonographers.

Two ultrasound fellowship-trained sonographers with prior experience in the use of ultrasound for ETT confirmation performed the assessments. A Zonare Z.One PRO ultrasound machine with an L14–5 linear transducer was used for all of the assessments. For each intubation, the dynamic technique was performed first by one sonographer. Then, the ETT was left in position while the other sonographer performed the static technique. Sonographers performed assessments in an alternating sequence of dynamic and static techniques to reduce the risk of shortening the learning curve with one technique. Each sonographer would leave the room after performing the sonographic assessment, so that neither sonographer was in the same room at the same time.

**Population Health Research Capsule**

What do we already know about this issue?

Ultrasound is increasingly being used to confirm endotracheal tube (ETT) location. However, there are variations in the techniques used.

What was the research question?

This study compared the static with the dynamic sonographic technique to determine which was more accurate for ETT identification.

What was the major finding of the study?

Both the static and dynamic ultrasound techniques were equally rapid and accurate for confirming ETT location.

How does this improve population health?

Either the dynamic or static technique may be used for ETT confirmation. Further studies are recommended in ED patients and with more-novice sonographers.

**Figure 1:**
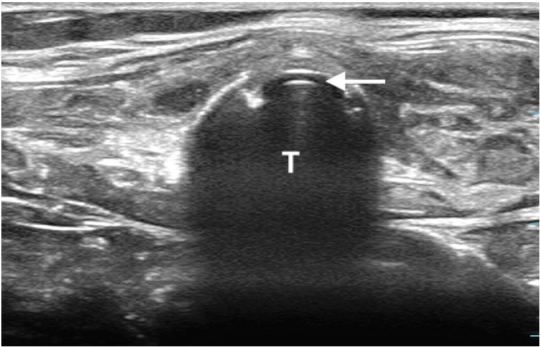
Endotracheal intubation using the static technique to confirm placement. *T*, trachea; white arrow, endotracheal tube.

**Figure 2:**
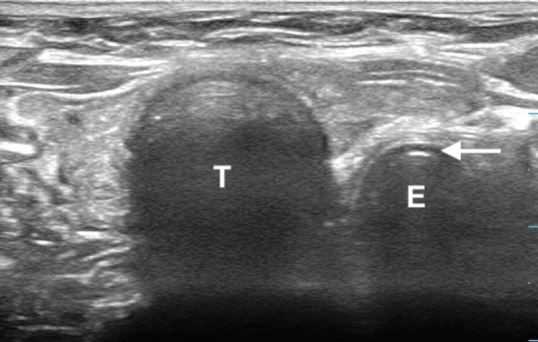
Esophageal intubation imaged with the static technique. *T*, trachea; *E*, esophagus; white arrow, endotracheal tube.

For the dynamic technique, sonographers placed the ultrasound transducer across the neck at the suprasternal level to locate the trachea and surrounding tissues.^6^ Visualization of motion artifact within the trachea confirmed tracheal intubation (Video 1). Visualization of a “second trachea” lateral to the true trachea confirmed esophageal intubation (Video 2). For the static technique, sonographers placed the transducer in the same location post-intubation, while the intubator gently rotated the tube side-to-side to create a motion artifact (Figure 1, Video 3).^7^ Presence of movement within the trachea confirmed tracheal intubation, while visualization of the “second trachea” confirmed esophageal intubation (Figure 2, Video 4).

A research assistant recorded the sonographer’s prediction of the ETT location, time to ETT prediction, and operator level of confidence after each intubation. Operator confidence was assessed using a Likert scale ranging from 1–5 with 1 signifying “not confident at all” and 5 signifying “very confident.” We performed a comparison between the predicted and actual location after study completion.

With an estimated 120 readings each for static and dynamic techniques, 95% level of significance, and a moderate effect size (0.3), the expected power for the study was above 90%. We used Microsoft Excel and SPSS statistical software to conduct the analysis. We used descriptive statistics, chi-square test, and t-test to analyze the relationships between the ultrasound static and dynamic techniques with respect to the accuracy of correctly identifying location of intubation, operator time to identification, and operator confidence. In addition, we included moderating variables such as operators, cadaver number, and actual location of the intubations in the analysis.

## RESULTS

A total of 120 intubations were performed. Each intubation was assessed with both the static and dynamic techniques, resulting in 240 total assessments. There were 62 tracheal intubations and 58 esophageal intubations. The static technique was 93.6% (95% confidence interval [CI] [84.3% to 98.2%]) sensitive and 98.3% specific (95% CI [90.8% to 99.9%]) for endotracheal confirmation (Table 1). The dynamic technique was 92.1% (95% CI [82.4% to 97.4%]) sensitive and 91.2% specific (95% CI [80.7% to 97.1%]%) for endotracheal confirmation (Table 2). There was no statistically significant difference in correctly identifying the location of the ETT between the static and dynamic ultrasound techniques. For the mean operator time to identification, there was no statistical difference between the static (6.72 seconds; 95% CI [5.53 to 7.9] seconds) and the dynamic (6.4 seconds; 95% CI [5.65 to 7.16] seconds) techniques. The mean operator confidence was not statistically different between the static (4.9/5.0; 95% CI [4.83 to 4.97]) and the dynamic (4.86/5.0; 95% CI [4.78 to 4.94]) techniques.

## DISCUSSION

In the ED setting, it is essential to quickly and accurately confirm correct ETT placement. While there are many options for confirmation, each has its own limitations. In fact, even colorimetric capnography may have false positives and negatives, resulting in an accuracy as low as 67.9% during cardiac arrest.^1^,^8^,^9^ Ultrasound has been suggested to be particularly valuable in this application due to the ability to rapidly identify ETT location without requiring ventilations and the subsequent risk of gastric distention and aspiration if the ETT is incorrectly placed. However, current studies have used a variety of techniques, with some relying upon a static assessment, while others use dynamic assessments.^2^–^4^,^7^,^10^–^11^

This is one of the first studies to directly compare static with dynamic ultrasound for the identification of ETT location, demonstrating no statistically significant difference between techniques. This is an important finding, as there has been concern that performing dynamic sonography for ETT confirmation may be more challenging because it requires more than one trained provider to be available to perform the confirmation.^7^ This may prevent the use of this technique in locations where only one ultrasound-trained provider is present. By twisting the ETT in one’s fingers post-intubation, the provider is able to replicate the dynamic technique without the need for a second provider.

Additionally, with the dynamic technique, placement is best assessed as the ETT is being inserted, and localization may be more limited if the ETT is not immediately identified during the intubation attempt. Finally, having the ultrasound probe on the neck may make the intubation attempt more difficult by providing extra pressure on the trachea and distorting upper airway anatomy. Alternatively, by performing the technique post-intubation, the neck remains unencumbered, thereby allowing the intubating provider to also perform external laryngeal manipulation if needed.

Interestingly, we found no difference in the confirmation time or operator confidence. Both studies were completed in an average of six seconds, which allowed for rapid confirmation with minimal risk of desaturation. Additionally, this examination could be performed while capnography was being obtained, with both confirmatory methods used to support each other in equivocal cases. Operator confidence was high with both techniques, suggesting that both providers felt comfortable with their assessments, which is an important finding in ultrasound studies because, if the operator is not confident in their assessment, they will be unlikely to use the examination clinically.

## LIMITATIONS

It is important to consider several limitations with respect to this study. First, it was performed in a cadaver model, which may not fully reflect the characteristics of a live patient. However, cadaver models have been used extensively for the evaluation of ultrasound for ETT confirmation and have demonstrated similar test characteristics to live patients for this modality.^7^,^11^–^13^ Additionally, we used only three cadavers in the study and it is possible this may not have fully represented the wider population. However, we intentionally used cadavers with significant differences in anatomy to best represent the variation in a larger population.

It is possible that the repeat intubations may have improved the accuracy of the sonographers due to increased practice. To avoid this we alternated cadavers and techniques between each use to reduce the potential for improving each sonographer’s learning curve during the study. While it is not possible to completely exclude the potential for sonographers to have improved their accuracy throughout the study, this was not supported by the data as equivalent numbers of misidentified ETT placements occurred in the early and later intubations. There is also no reason to suggest that this would differentially affect one technique over another. Moreover, this study was designed to evaluate the test characteristics of dynamic vs. static sonography for ETT localization. Therefore, it is important to ensure similar rates of tracheal and esophageal intubation, which would not be possible in an ED setting due to low overall rates of esophageal intubation.^14^ Because this study was performed by two sonographers with prior experience using ultrasound for ETT confirmation, it is possible that the results may have differed if less experienced sonographers were used. However, the use of ultrasound for ETT confirmation has been suggested to have a rapid learning curve.^15^ Nonetheless, further studies are advised to determine whether the accuracy of static vs. dynamic techniques differs in less experienced providers.

## CONCLUSION

This study demonstrated that the static and dynamic sonographic approaches to confirming endotracheal intubation were both rapid and accurate with no significant difference between modalities. Further studies are recommended to compare these techniques in ED patients and with more novice sonographers.

## Figures and Tables

**Video 1:** Endotracheal intubation with the dynamic technique.

**Video 2:** Esophageal intubation with the dynamic technique.

**Video 3:** Endotracheal intubation with the static technique.

**Video 4:** Esophageal intubation with the static technique.

**Table 1:** Accuracy of the static technique for endotracheal intubation.

	Endotracheal intubation	Esophageal intubation	Total
Endotracheal location on ultrasound	58	1	59
Esophageal location on ultrasound	4	57	61
Total	62	58	

**Table 2:** Accuracy of the dynamic technique for endotracheal intubation.

	Endotracheal intubation	Esophageal intubation	Total
Endotracheal location on ultrasound	58	5	63
Esophageal location on ultrasound	5	52	57
Total	62	58	
